# Supplementary material for: microRNA Expression of Renal Proximal Tubular Epithelial Cells and Their Extracellular Vesicles in an Inflammatory Microenvironment In Vitro
Source: Int J Mol Sci. 2023 Jul 4;24(13):11069. doi: 10.3390/ijms241311069 (PMC10341938; doi:10.3390/ijms241311069)

Suppl. Table S1: Regulated miRNAs in PTC. The miRNAs and piRNAs with a minimum log2FC of > ±0.75 and an adjusted p value (p-adj) of less than 0.1.

Name	Accession	Stimulation/Ctrl (log2FC)	p-adj
HSA-MIR-99B-5P	MIMAT0038112	7,812	0,001
HSA-MIR-146A-3P	MIMAT0004608	4,778	0,000
HSA-MIR-147B	MIMAT0004928, MIMAT0037331	4,244	0,000
HSA-MIR-100-3P	MIMAT0004512	4,051	0,004
HSA-MIR-146A-5P	MIMAT0000449	3,384	0,000
HSA_PIR_020490	DQ598167	3,195	0,010
HSA_PIR_018573	DQ595539	3,151	0,028
HSA_PIR_020381	DQ597997	3,080	0,017
HSA_PIR_017936	DQ594740	2,966	0,016
HSA-MIR-125B-1-3P	MIMAT0004592	2,768	0,089
HSA-MIR-155-5P	MIMAT0000646	2,438	0,000
HSA-MIR-1246	MI0006381	1,632	0,089
HSA_PIR_001312	DQ571813	1,132	0,035
HSA_PIR_020009	DQ597484	-1,194	0,099
HSA_PIR_017033	DQ593538	-1,490	0,037
HSA-MIR-140-3P	MIMAT0004597	-1,505	0,016
HSA-MIR-744-5P	MIMAT0004945	-1,711	0,076
HSA-MIR-335-5P	MIMAT0000765	-1,872	0,006
HSA-MIR-296-3P	MIMAT0004679	-2,027	0,037
HSA_PIR_020815	DQ598651	-2,440	0,007
HSA-MIR-186-5P	MIMAT0000456	-8,608	0,000
HSA-MIR-210-3P	MIMAT0000267	-8,773	0,000
HSA-MIR-128-3P	MIMAT0000424	-9,909	0,000

Suppl. Table S2: Regulated miRNAs in EVs. The miRNAs and piRNAs with a minimum log2FC of > ±0.75 and an adjusted p value (p-adj) of less than 0.1.

Name	Accession	Stimulation/Ctrl (log2FC)	p-adj
HSA-MIR-147B	MIMAT0004928, MIMAT0037331	3,007	0,005
HSA_PIR_014635	DQ590029	2,204	0,080
HSA-MIR-3613-5P	MIMAT0017990	2,163	0,044
HSA_PIR_019179	DQ596327	1,987	0,084
HSA_PIR_000805	DQ571003	1,973	0,000
HSA-MIR-146A-5P	MIMAT0000449	1,944	0,000
HSA_PIR_016659	DQ592932	1,815	0,000
HSA_PIR_016735	DQ593039	1,748	0,000
HSA_PIR_020381	DQ597997	1,659	0,013
HSA_PIR_016658	DQ592931	1,529	0,000
HSA-MIR-320C	MIMAT0005793	1,336	0,019
HSA-MIR-155-5P	MIMAT0000646	1,232	0,000
HSA-MIR-141-3P	MIMAT0000432	1,025	0,000

HSA-MIR-23B-3P	MIMAT0000418	0,954	0,001
HSA-MIR-221-3P	MIMAT0000278	0,801	0,000
HSA-MIR-148A-3P	MIMAT0000243	0,764	0,084
HSA_PIR_018570	DQ595536	-0,882	0,031
HSA_PIR_009228	DQ582496	-0,883	0,026
HSA_PIR_001312	DQ571813	-1,226	0,000
HSA_PIR_004308	DQ575882	-1,259	0,091
HSA_PIR_009981	DQ583434	-1,346	0,061
HSA_PIR_006426	DQ578783	-1,378	0,001
HSA_PIR_004307	DQ575881	-1,580	0,023
HSA_PIR_017724	DQ594465	-1,661	0,000
HSA_PIR_019951	DQ597403	-1,701	0,000
HSA_PIR_020541	DQ598252	-1,736	0,011
HSA_PIR_004801	DQ576605	-1,754	0,098
HSA-MIR-143-3P	MIMAT0000435	-1,851	0,098
HSA_PIR_020485	DQ598159	-1,946	0,001
HSA_PIR_001318	DQ571823	-2,218	0,000
HSA_PIR_020829	DQ598677	-2,256	0,000
HSA_PIR_015026	DQ590548	-2,271	0,000
HSA_PIR_020496	DQ598175	-2,357	0,000
HSA_PIR_019420	DQ596670	-2,410	0,024
HSA_PIR_019914	DQ597347	-2,534	0,000
HSA_PIR_004153	DQ575660	-2,802	0,000
HSA_PIR_017716	DQ594453	-2,872	0,000
HSA_PIR_019752	DQ597110	-2,954	0,000
HSA_PIR_017723	DQ594464	-3,023	0,011
HSA_PIR_020497	DQ598177	-3,044	0,000
HSA_PIR_000794	DQ570992	-3,156	0,002
HSA_PIR_001311	DQ571812	-3,217	0,011
HSA_PIR_020391	DQ598016	-3,431	0,000
HSA_PIR_019825	DQ597218	-3,739	0,000
HSA-MIR-3687	MIMAT0018115	-3,836	0,000
HSA_PIR_020326	DQ597916	-3,888	0,000
HSA_PIR_016677	DQ592953	-3,915	0,000
HSA_PIR_000765	DQ570956	-4,118	0,000
HSA_PIR_020499	DQ598183	-4,386	0,000
HSA_PIR_011300	DQ585261	-4,789	0,000

Suppl. Figure S1: KEGG-pathway analysis of the regulated miRNAs in PTC and EVs. KEGG-pathway analysis of miR-146a-5p, miR-147b, and miR-155-5p based on the miRNA signature performed with miRNet (miRTarBase v8.0).

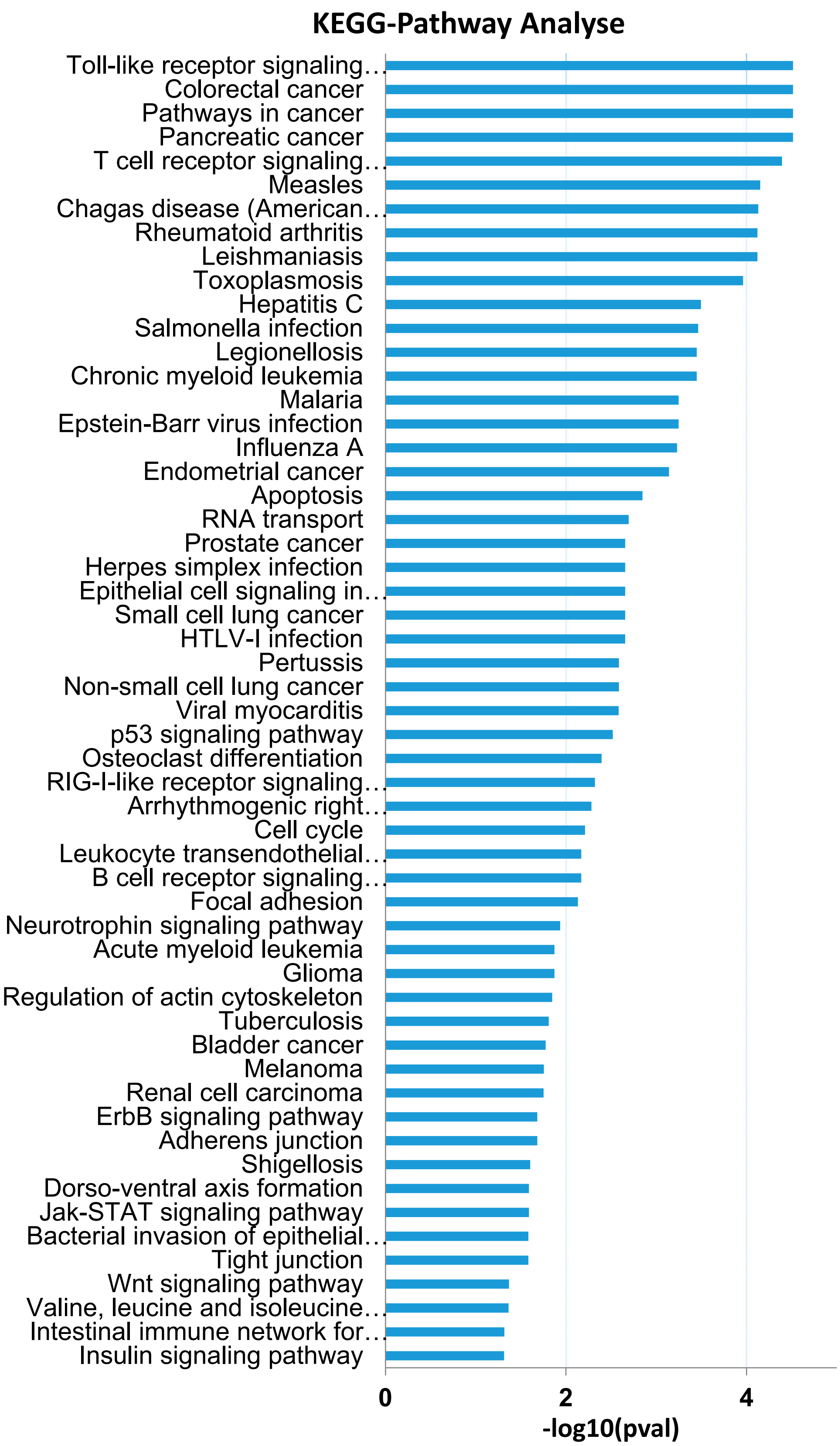

Supplement: Supplementary file 1 [file ijms-24-11069-s001.zip › ijms-2480137-supplementary.pdf]
